# Supplementary material for: Evolutionary games with environmental feedbacks
Source: Nat Commun. 2020 Feb 14;11:915. doi: 10.1038/s41467-020-14531-6 (PMC7021758; doi:10.1038/s41467-020-14531-6)
Supplement: Supplementary file 1 — Supplementary Information [file 41467_2020_14531_MOESM1_ESM.pdf]

Supplementary Information for

"Evolutionary games with environmental feedbacks"

Andrew R. Tilman, Joshua B. Plotkin, and Erol Akçay

Contents

Supplementary Note 1: Renewable resource model **2**
1.1 Renewable resource stability analysis . . . 3
1.2 Renewable resource system-level analysis . . . 5

Supplementary Note 2: Decaying resource model **6**
2.1 Decaying resource stability analysis . . . 8
2.2 Decaying resource system-level analysis . . . 9

Supplementary Note 3: The existence of cycles **9**

Supplementary Note 4: Cognitive strategy dynamics as an eco-evolutionary game with decaying resource **11**
4.1 Analysis . . . 12
4.2 Application to Rand et al. model . . . 14

Supplementary Note 5: Common-pool resource harvesting **14**

Supplementary Note 6: Market pricing CPR model **16**
6.1 Market pricing analysis . . . 17

Supplementary Note 7: Frequency dependent harvesting efficiency CPR model **18**

Supplementary Note 8: Environmental feedback with tipping points **23**
8.1 Model of Weitz et al. . . . 24
8.2 Simple tipping point model . . . 25
8.3 Environments with a distribution of tipping points . . . 25
8.4 Linear case . . . 27

Supplementary Note 9: Relationship to Sigdel et al. **28**

Supplementary References **31**

Supplementary Note 1: Renewable resource model

We analyze a two-strategy evolutionary-game-theoretic model that incorporates environmental feedbacks, governed by renewable resource dynamics. Suppose that there is a resource stock, m, that in the absence of consumption or harvest pressure grows logistically, and is diminished through harvesting or consumption that is associated with the strategies in a game. For a 2-strategy game, let e_L and e_H be the harvest effort of strategies L and H, respectively, where we assume, that $e_L < e_H$. The dynamics of m are governed by

$$\frac{dm}{dt} = rm\left(1 - \frac{m}{k}\right) - qm\left(e_L x + e_H(1-x)\right) \tag{1}$$

where x is the fraction of the population playing strategy L, r is the intrinsic rate of growth, k is the carrying capacity of m, and q is a parameter that maps resource degradation pressures (or harvesting efforts) (e_L, e_H) onto the rate of reduction in the resource. We assume that environmental impact rates are restricted so that m will be positive at equilibrium. This implies that $e_H \in (0, r/q)$ and $e_L \in [0, e_H)$.

Let $n \in [0,1]$ be a normalized measure of the state of the environment that maps onto the payoff structure of the game. We relate m to n with the linear relationship

$$n = \frac{m - k\left(1 - \frac{qe_H}{r}\right)}{(e_H - e_L)\frac{qk}{r}} \tag{2}$$

so that when the whole population chooses strategy L, corresponding to harvesting pressure e_L, the equilibrium value of m maps to the environmental metric $n = 1$. Similarly, if the whole population adheres to strategy H, with e_H environmental pressure, $n \to 0$.

Next, suppose that the state of the environment influences the payoffs of the game. We use a payoff matrix for a 2-strategy game with payoffs that are dependent on the normalized environmental measure, n, given by

$$\Pi(n) = (1-n)\begin{bmatrix} R_0 & S_0 \\ T_0 & P_0 \end{bmatrix} + n\begin{bmatrix} R_1 & S_1 \\ T_1 & P_1 \end{bmatrix} \tag{3}$$

so that the matrix entries correspond to the payoffs of the game under conditions of a poor or rich environmental state. We can write the payoff for playing strategy L and strategy H as

$$\pi_L(x,n) = (1-n)(R_0 x + S_0(1-x)) + n(R_1 x + S_1(1-x)) \tag{4}$$

$$\pi_H(x,n) = (1-n)(T_0 x + P_0(1-x)) + n(T_1 x + P_1(1-x)). \tag{5}$$

Following the replicator equation, the payoffs from the game determine the evolution of the fractions of the population that play each strategy. In all, we can write our system as

$$\epsilon \dot{x} = x(1-x)(\pi_L(x,n) - \pi_H(x,n)) \tag{6}$$

$$\dot{n} = \left(r - q(e_L n + e_H(1-n))\right)(x - n). \tag{7}$$

We let $\tau = t/\epsilon$ and re-scale time so that our system can be written as

$$x' = x(1-x)(\pi_L(x,n) - \pi_H(x,n)) \tag{8}$$

$$n' = \epsilon\left(r - q(e_L n + e_H(1-n))\right)(x - n). \tag{9}$$

where $\frac{dx}{d\tau} = x'$.

1.1 Renewable resource stability analysis

This model has up to four equilibria within the state space. We have two edge equilibria, at $(x^*, n^*) \in \{(0,0), (1,1)\}$ and up to two interior equilibria that occur when $\pi_L(x,n) = \pi_H(x,n)$ and $n = x$. While there are up to two points that meet the criterion for a potential interior equilibrium, they need not fall inside the state space. Also, note that while $n' = 0$ at $n = \frac{e_H - r/q}{e_H - e_L}$ this too falls outside of our state space since we assume that $e_H \in (0, r/q)$ and $e_L \in (0, e_H)$.

At the edge equilibria, stability conditions are simple. At $(x^*, n^*) = (0,0)$, stability occurs if and only if $P_0 > S_0$. At At $(x^*, n^*) = (1,1)$, stability occurs if and only if $R_1 > T_1$.

Next, we analyze the conditions for stability of interior equilibria. The interior equilibrium, defined by $\pi_L(x,n) = \pi_H(x,n)$ and $x = n$ occurs when

$$n = \frac{(S_0 - P_0)(1-x) + (R_0 - T_0)x}{(P_1 - S_1 + S_0 - P_0)(1-x) + (T_1 - R_1 + R_0 - T_0)x} \tag{10}$$

and $x = n$. We can substitute and solve for the equilibrium level of the environmental indicator and the equilibrium strategy fraction, which can be streamlined by defining four values as

$$\Delta_L^1 = \pi_H(1,1) - \pi_L(1,1) = T_1 - R_1 \tag{11}$$

$$\Delta_H^1 = \pi_H(0,1) - \pi_L(0,1) = P_1 - S_1 \tag{12}$$

$$\delta_L^0 = \pi_L(1,0) - \pi_H(1,0) = R_0 - T_0 \tag{13}$$

$$\delta_H^0 = \pi_L(0,0) - \pi_H(0,0) = S_0 - P_0 \tag{14}$$

where the Δ's correspond to the incentive to switch to the high impact strategy and the δ's correspond to the incentive to switch to the low impact strategy. The superscripts correspond to the environmental state and the subscripts denote the resident strategy in the population.

Using these δ's and Δ's allows us to write the interior equilibrium location as

$$x_\pm^* = n_\pm^* = \frac{2\delta_H^0 + \Delta_H^1 - \delta_L^0 \pm \sqrt{(\Delta_H^1 - \delta_L^0)^2 + 4\Delta_L^1 \delta_H^0}}{2(\delta_H^0 + \Delta_H^1 - \delta_L^0 - \Delta_L^1)}. \tag{15}$$

Local stability at such an equilibrium can be computed from the Jacobian matrix evaluated at an interior equilibrium, which is

$$J^* = \begin{bmatrix} x(1-x)\frac{\partial g}{\partial x}(x,n) & x(1-x)\frac{\partial g}{\partial n}(x,n) \\ \epsilon\,(r - q(e_L n + e_H(1-n))) & -\epsilon\,(r - q(e_L n + e_H(1-n))) \end{bmatrix}_{(x,n)=(x^*,n^*)} \tag{16}$$

where $g(x,n) = \pi_L(x,n) - \pi_H(x,n)$. Stability requires $\mathrm{Det}(J^*) > 0$ and $\mathrm{Tr}(J^*) < 0$.

First, consider the determinant of the Jacobian matrix,

$$\mathrm{Det}(J^*) = -\epsilon\,(r - q(e_L n^* + e_H(1-n^*)))\, x^*(1-x^*) \left(\frac{\partial g}{\partial n}(x^*, n^*) + \frac{\partial g}{\partial x}(x^*, n^*) \right). \tag{17}$$

Due to the restrictions on the level of environmental impact, $e_H \in (0, r/q)$ and $e_L \in [0, e_H)$, and the fact that both x and n are between zero and one, we can conclude that $\mathrm{Det}(J^*) > 0$ if and only if $\frac{\partial g}{\partial n}(x^*, n^*) + \frac{\partial g}{\partial x}(x^*, n^*) < 0$. The terms of interest are

$$\frac{\partial g}{\partial n}(x^*,n^*) = -\left[(\Delta_H^1+\delta_H^0)(1-x^*)+(\delta_L^0+\Delta_L^1)x^*\right] \tag{18}$$

$$\frac{\partial g}{\partial x}(x^*,n^*) = (\delta_L^0-\delta_H^0)(1-n^*)+(\Delta_H^1-\Delta_L^1)n^* \tag{19}$$

Recalling that at an interior equilibrium $x^* = n^*$ we can simplify the expression for a positive determinant of the Jacobian matrix to

$$\delta_L^0-\Delta_H^1-2\delta_H^0+2(\Delta_H^1-\delta_L^0-\Delta_L^1+\delta_H^0)x^*<0 \tag{20}$$

Evaluation at the equilibria yields the condition for a positive determinant, and is

$$\pm\sqrt{(\Delta_H^1-\delta_L^0)^2+4\Delta_L^1\delta_H^0}<0. \tag{21}$$

Where $\sqrt{(\Delta_H^1-\delta_L^0)^2+4\Delta_L^1\delta_H^0}<0$ corresponds to x_+^* and $-\sqrt{(\Delta_H^1-\delta_L^0)^2+4\Delta_L^1\delta_H^0}<0$ corresponds to x_-^*.

The trace of the Jacobian matrix is

$$\mathrm{Tr}(J^*) = x^*(1-x^*)\frac{\partial g}{\partial x}(x^*,n^*)-\epsilon\left(r-q(e_Ln^*+e_H(1-n^*))\right) \tag{22}$$

where $g(x,n)=\pi_L(x,n)-\pi_H(x,n)$. Given $\mathrm{Det}(J^*)>0$, stability at an interior equilibrium will occur if and only if $\mathrm{Tr}(J^*)<0$. A sufficient condition for a negative trace is $\frac{\partial g}{\partial x}(x^*,n^*)<0$, because this implies that both terms of the trace are negative. This occurs when either

$$\Delta_H^1+\delta_L^0<0 \tag{23}$$

or

$$\delta_L^0\Delta_H^1<\Delta_L^1\delta_H^0. \tag{24}$$

On the other hand if Supplementary Conditions 23 and 24 are violated, stability is still possible but not assured. In this scenario, stability depends on the relative speed of environmental feedbacks. In this case, stability occurs at the interior equilibrium when

$$\epsilon>\epsilon_{\text{crit}} = \frac{\begin{aligned}&\left(\sqrt{4\Delta_L^1\delta_H^0+(\Delta_H^1-\delta_L^0)^2}-\Delta_H^1-\delta_L^0\right)\\ &\times\left(\sqrt{4\Delta_L^1\delta_H^0+(\Delta_H^1-\delta_L^0)^2}-2\Delta_L^1+\Delta_H^1-\delta_L^0\right)\\ &\times\left(\sqrt{4\Delta_L^1\delta_H^0+(\Delta_H^1-\delta_L^0)^2}-\Delta_H^1+\delta_L^0-2\delta_H^0\right)\end{aligned}}{\begin{aligned}&4(\Delta_L^1-\Delta_H^1+\delta_L^0-\delta_H^0)\\ &\times\Big[q((\Delta_H^1-\delta_L^0)(e_H+e_L)-2(\Delta_L^1e_H-\delta_H^0e_L))\\ &+q(e_H-e_L)\sqrt{4\Delta_L^1\delta_H^0+(\Delta_H^1-\delta_L^0)^2}\\ &+2r(\Delta_L^1-\Delta_H^1+\delta_L^0-\delta_H^0)\Big]\end{aligned}} \tag{25}$$

where ϵ is the speed of environmental feedbacks relative to strategy updating. As r increases, the region of the parameter space that leads to a stable interior equilibrium increases. This makes sense, both ϵ and r increase the speed of environmental dynamics, and thus changes in these parameters have similar effects on outcomes.

1.2 Renewable resource system-level analysis

The x_+^* equilibrium is an element of the unit interval when the following conditions hold:

$$x_+^* \in (0,1) \iff \begin{cases} [\delta_H^0 + \Delta_H^1 - \delta_L^0 - \Delta_L^1 > 0 \text{ and} \\ (\delta_H^0 < 0 \text{ or } \Delta_H^1 - \delta_L^0 + 2\delta_H^0 > 0) \text{ and} \\ (\Delta_L^1 < 0 \text{ and } \Delta_H^1 - \delta_L^0 - 2\Delta_L^1 > 0)] \\ \text{or} \\ [\delta_H^0 + \Delta_H^1 - \delta_L^0 - \Delta_L^1 < 0 \text{ and} \\ (\delta_H^0 < 0 \text{ and } \Delta_H^1 - \delta_L^0 + 2\delta_H^0 < 0) \text{ and} \\ (\Delta_L^1 < 0 \text{ or } \Delta_H^1 - \delta_L^0 - 2\Delta_L^1 < 0)] \end{cases} \tag{26}$$

Similarly, the x_-^* equilibrium is an element of the unit interval when the following conditions hold:

$$x_-^* \in (0,1) \iff \begin{cases} [\delta_H^0 + \Delta_H^1 - \delta_L^0 - \Delta_L^1 > 0 \text{ and} \\ (\delta_H^0 > 0 \text{ and } \Delta_H^1 - \delta_L^0 + 2\delta_H^0 > 0) \text{ and} \\ (\Delta_L^1 > 0 \text{ or } \Delta_H^1 - \delta_L^0 - 2\Delta_L^1 > 0)] \\ \text{or} \\ [\delta_H^0 + \Delta_H^1 - \delta_L^0 - \Delta_L^1 < 0 \text{ and} \\ (\delta_H^0 > 0 \text{ or } \Delta_H^1 - \delta_L^0 + 2\delta_H^0 < 0) \text{ and} \\ (\Delta_L^1 > 0 \text{ and } \Delta_H^1 - \delta_L^0 - 2\Delta_L^1 < 0)] \end{cases} \tag{27}$$

Ascertaining what these criteria mean for system-level properties of interest is simplified by considering four cases that span the values of Δ_L^1 and δ_H^0.

Case 1: ($\Delta_L^1 < 0$ and $\delta_H^0 < 0$) In this case, both edge equilibria are stable and only an interior saddle equilibrium, given by x_+^* falls in the interior of the state space. Thus, this corresponds to bistability of strategy L and H where the resulting environmental and strategy state depends on the initial environmental and strategy conditions. This result is invariant to the details of the environmental feedback timescale or the payoff structure of the game (other than the restriction that $\Delta_L^1 < 0$ and $\delta_H^0 < 0$).

Case 2: ($\Delta_L^1 > 0$ and $\delta_H^0 > 0$) In this case both edge equilibria are unstable, and only the x_-^* equilibrium is in the state space. The stability analysis indicates that x_-^* is guaranteed to be a locally stable equilibrium when either $\Delta_H^1 + \delta_L^0 < 0$ or $\Delta_H^1 \delta_L^0 < \Delta_L^1 \delta_H^0$. If both of these conditions are violated, then stability will be governed by the condition on ϵ. When the interior equilibrium is stable, we find no evidence of limit cycles and expect all initial conditions to lead to the interior equilibrium. When it is unstable, limit cycles will result from all starting conditions. These are the cases that correspond to the 'oscillating tragedy of the commons' in Weitz et al. [1].

Case 3: **($\Delta_L^1 < 0$ and $\delta_H^0 > 0$)** In this case, both interior equilibria can fall within the state space, given all the following hold:

$$\begin{cases} |\Delta_H^1 - \delta_L^0| > 2\sqrt{-\Delta_L^1 \delta_H^0} \\ \Delta_H^1 - \delta_L^0 > \Delta_L^1 - \delta_H^0 \\ \Delta_H^1 - \delta_L^0 > -2\delta_H^0 \\ \Delta_H^1 - \delta_L^0 > 2\Delta_L^1 \end{cases} \tag{28}$$

For these four conditions to hold, given $\Delta_L^1 < 0$ and $\delta_H^0 > 0$, we need either $\Delta_H^1 - \delta_L^0 > 2\sqrt{-\Delta_L^1 \delta_H^0}$, or $\Delta_H^1 - \delta_L^0 < -2\sqrt{-\Delta_L^1 \delta_H^0}$. The latter cannot hold simultaneously with the three other conditions, since $-2\sqrt{-\Delta_L^1 \delta_H^0}$ must be less that at least one of the other terms that set minimums on $\Delta_H^1 - \delta_L^0$. Further, the right-hand-side of the first inequality is positive, while the rest are negative. Thus all four of the conditions hold if and only if $\Delta_H^1 - \delta_L^0 > 2\sqrt{-\Delta_L^1 \delta_H^0}$. Also, since violating the first condition implies that interior equilibria cannot exist, there will either be two or zero interior equilibria (with a special case where the nullclines are tangent at one point). When there is no interior equilibrium, dynamics will tend toward the state where all individuals employ the strategy with low environmental impact. This state is stable, since $\Delta_L^1 < 0$. When there are two interior equilibria, one will be a saddle, and one will be stable or unstable depending on parameters and the degree of timescale separation. If this interior equilibrium is stable, then the resulting dynamics of the system will be similar to the bi-stable regime described above. If this interior equilibrium is unstable, the system may have limit cycles from some initial conditions and tend toward dominance of the low impact strategy from other initial conditions. It is also possible, for low values of ϵ, that a limit cycle will not exist, and instead dynamics will tent toward the low impact state from all initial conditions.

Case 4: **($\Delta_L^1 > 0$ and $\delta_H^0 < 0$)** Lastly, following similar arguments as in case 3, we conclude that two interior equilibria will result when $\Delta_H^1 - \delta_L^0 < -2\sqrt{-\Delta_L^1 \delta_H^0}$, and no interior equilibria will occur if $\Delta_H^1 - \delta_L^0 > -2\sqrt{-\Delta_L^1 \delta_H^0}$. In this case, the edge equilibrium where the high impact strategy dominates will always be stable. If there are two interior equilibria, then as in case 3, depending on the value of ϵ, either bi-stability, limit cycles embedded in a bi-stable regime, or dominance of the high impact strategy from all initial conditions will result.

Supplementary Note 2: Decaying resource model

While some resources are intrinsically self-renewing, many others are intrinsically decaying, and are maintained by production as a consequence of agents' strategies. Here, we treat this case.

Let m be the concentration a resource that impacts the payoffs of players in a game, and is created as a byproduct playing the game. We assume that in the absence of production by players of the game, the concentration of m decays exponentially. We also assume that strategy L has a low emissions rate of the resource, e_L, and strategy H has a higher emissions

rate rate, e_H. Given these assumptions we can model the dynamics of m as

$$\frac{dm}{dt} = -\alpha m + e_L x + e_H(1-x) \tag{29}$$

where x is the fraction of the population that employs strategy L, and $(1-x)$ is the fraction of strategy H players. While m can be any resource that meets the assumptions above, a clear example with societal relevance is pollution. Many actions generate pollution, and stocks of pollution impact many systems. However, there is a more broad class of problems that also fit within this framework, including the cognition-environment feedbacks studied by Rand et al. [2].

As in the case of the regenerating resource studied in the previous section, we define a metric of the environmental state, n,

$$n = \frac{e_H - \alpha m}{e_H - e_L} \tag{30}$$

such that when the whole population has high emissions, e_H, $n \to 0$ and when the whole population has low emissions, e_L, $n \to 1$. Whereas m is a direct measure of the concentration of a resource stock, for example a pollutant, n is a normalized transformation of m that is used to write the payoff structure of the game being considered. Employing a change of variables, the dynamics of n can be modeled as

$$\dot{n} = \alpha(x-n). \tag{31}$$

We use the same payoff matrix as before for the 2-strategy game with payoffs that are dependent on the normalized environmental measure, n, given by

$$\Pi(n) = (1-n)\begin{bmatrix} R_0 & S_0 \\ T_0 & P_0 \end{bmatrix} + n\begin{bmatrix} R_1 & S_1 \\ T_1 & P_1 \end{bmatrix} \tag{32}$$

so that the matrix entries correspond to the payoffs of the game under conditions of a poor or rich environmental state. We can write the payoff for playing strategy L and strategy H as

$$\pi_L(x,n) = (1-n)(R_0 x + S_0(1-x)) + n(R_1 x + S_1(1-x)) \tag{33}$$
$$\pi_H(x,n) = (1-n)(T_0 x + P_0(1-x)) + n(T_1 x + P_1(1-x)) \tag{34}$$

when x is the fraction of the population that plays strategy L and has low emissions. Following the replicator equation, the payoffs from the game determine the evolution of the proportion of the population that plays each strategy. In all, we can write our system as

$$\epsilon\dot{x} = x(1-x)(\pi_L(x,n) - \pi_H(x,n)) \tag{35}$$
$$\dot{n} = \alpha(x-n). \tag{36}$$

Let $\tau = t/\epsilon$ to re-scale time so that our system can be written as

$$x' = x(1-x)(\pi_L(x,n) - \pi_H(x,n)) \tag{37}$$
$$n' = \epsilon\alpha(x-n) \tag{38}$$

where $\frac{dx}{d\tau} = x'$.

2.1 Decaying resource stability analysis

This model also has up to four equilibria within the state space. We have 2 edge equilibria, at $(x^*, n^*) \in \{(0,0),(1,1)\}$ and up to two interior equilibria that occur when $\pi_L(x,n) = \pi_H(x,n)$ and $n = x$.

We analyze the Jacobian matrix to derive the conditions for stability of interior equilibria. The Jacobian matrix for this system at an interior equilibrium is

$$J^* = \begin{bmatrix} x(1-x)\frac{\partial g}{\partial x}(x,n) & x(1-x)\frac{\partial g}{\partial n}(x,n) \\ \epsilon\alpha & -\epsilon\alpha \end{bmatrix}_{(x,n)=(x^*,n^*)} \tag{39}$$

where, once again, $g(x,n) = \pi_L(x,n) - \pi_H(x,n)$. Also, the location of the interior equilibrium is the same as in the renewable resource model, with

$$\Delta_L^1 = T_1 - R_1 \tag{40}$$

$$\Delta_H^1 = P_1 - S_1 \tag{41}$$

$$\delta_L^0 = R_0 - T_0 \tag{42}$$

$$\delta_H^0 = S_0 - P_0 \tag{43}$$

used to simplify our expressions and take into account only the differences in the payoffs, without loss of generality. The simplified expression for the interior equilibrium is

$$n^* = x^* = \frac{2\delta_H^0 + \Delta_H^1 - \delta_L^0 \pm \sqrt{(\Delta_H^1 - \delta_L^0)^2 + 4\Delta_L^1\delta_H^0}}{2(\delta_H^0 + \Delta_H^1 - \delta_L^0 - \Delta_L^1)}. \tag{44}$$

In relation to the analyses for the renewable resource model, the structure of the Jacobian matrix for the decaying resource model is very similar. This will make our analysis easier because much of it carries over directly from previous sections.

Stability at an interior equilibrium can be determined by trace and determinant of the Jacobian matrix. As before, the determinant is positive if and only if

$$\frac{\partial g}{\partial n}(x^*, n^*) + \frac{\partial g}{\partial x}(x^*, n^*) < 0 \tag{45}$$

and a sufficient condition for a negative trace is

$$\frac{\partial g}{\partial x}(x^*, n^*) < 0. \tag{46}$$

However, if Supplementary Condition 46 is violated but Supplementary Condition 45 holds, stability is still possible, and will depend on the speed of environmental feedbacks. In this case, the condition for stability is

$$\epsilon\alpha > \epsilon_{\text{crit}}\alpha = \frac{\begin{array}{c}\left(\sqrt{4\Delta_L^1\delta_H^0 + (\Delta_H^1 - \delta_L^0)^2} - \Delta_H^1 - \delta_L^0\right) \\ \left(\sqrt{4\Delta_L^1\delta_H^0 + (\Delta_H^1 - \delta_L^0)^2} - 2\Delta_L^1 + \Delta_H^1 - \delta_L^0\right) \\ \left(\sqrt{4\Delta_L^1\delta_H^0 + (\Delta_H^1 - \delta_L^0)^2} - \Delta_H^1 + \delta_L^0 - 2\delta_H^0\right)\end{array}}{8(\Delta_L^1 - \Delta_H^1 + \delta_L^0 - \delta_H^0)^2} \tag{47}$$

so that fast enough environmental dynamics can stabilize any interior equilibrium, even if Supplementary Condition 46 is not met. Lastly, $g(x, n)$ is unchanged from the renewable resource model, implying that the forms of Supplementary Condition 46 and Supplementary Condition 45 also remain unchanged from the renewable resource model.

Now, we consider the edge equilibria. As before, at $(x^*, n^*) = (0, 0)$ stability occurs if and only if $\delta_H^0 = S_0 - P_0 < 0$. Again, $(x^*, n^*) = (1, 1)$ is stable if and only if $\Delta_L^1 = T_1 - R_1 < 0$. Thus if both Δ_L^1 and δ_H^0 are positive, then both edge equilibria are unstable.

2.2 Decaying resource system-level analysis

For the decaying resource model, the system-level analyses do not change from the renewable resource model, and thus the regions of the parameter space as divided in Figure 2do not change, and retain the same qualitative interpretations. This is because the position of the equilibria are unchanged and the sufficient conditions for stability of an interior equilibrium depend only on the δ parameters which share definitions in both systems. Thus, we can conclude that the only change to the system-level analysis from the renewable resource model is the value of ϵ_{crit} that divides cycles from a stable interior equilibrium in the regions of the parameter space where cycles can occur.

Supplementary Note 3: The existence of cycles

We alluded to the existence of limit cycles in some regions of the parameter space, and suggested that limit cycles do not occur in other regions of the parameter space. A challenge to making such claims arises because local stability analyses are not sufficient to describe global dynamics. In this section we detail the analysis on the existence, (or non-existence) of limit cycles in our framework. We break this analysis down into cases, following our local stability analysis.

Case 1: ($\Delta_L^1 < 0$ and $\delta_H^0 < 0$) We showed that in this case the only interior equilibrium point is a saddle. In two dimensional systems, a limit cycle must contain at least one equilibrium point. However, in this region, the interior equilibrium is a saddle point, which cannot be the only equilibrium point within a limit cycle in a two dimensional system. Therefore, we can conclude that this region of parameter space cannot contain limit cycles.

Case 2: ($\Delta_L^1 > 0$ and $\delta_H^0 > 0$) In this region only one equilibrium is present in the interior of the state space, and it is either stable or unstable, never a saddle point. Our local stability analysis indicated that when $\Delta_L^1 \delta_H^0 < \delta_L^0 \Delta_H^1$ then decreasing ϵ below ϵ_{crit} would destabilize the interior equilibrium, leading to super-critical Hopf bifurcation. Since in this setting there are no stable equilibria (in the interior or boundary of the state space) persistent oscillations result. Simulations indicate that these oscillations are stable limit cycles. While we can prove the existence of these oscillations for part a region of the parameter space, it is our goal to preclude them elsewhere.

Consider the region of the parameter space where $\Delta_L^1 \delta_H^0 > \delta_L^0 \Delta_H^1$. In this region, the slope of the strategy nullcline, is negative. Thus, we can see graphically that for a fixed value of n

dynamics converge to the strategy nullcline. This implies that when strategy dynamics are fast relative to environmental dynamics (small ϵ), the interior equilibrium is globally stable since dynamics quickly approach the strategy nullcline, then slowly converge to the interior equilibrium. Conversely, if environmental dynamics are fast, dynamics quickly converge to the environmental nullcline then slowly converge to the interior equilibrium (whether $\Delta_L^1 \delta_H^0 > \delta_L^0 \Delta_H^1$ holds or not). Therefore, we can conclude that if limit cycles exist in the region of the parameter space where $\Delta_L^1 \delta_H^0 > \delta_L^0 \Delta_H^1$, they must only occur for intermediate values of ϵ.

Limit cycles can be ruled out by the Dulac-Bendixson theorem. If we can find a suitable function $\varphi(x, n)$ such that

$$\frac{\partial(\varphi x')}{\partial x} + \frac{\partial(\varphi n')}{\partial n} < 0 \tag{48}$$

for all values of x and n in our state space, then we can rule out limit cycles for that region of the parameter space. Consider the Dulac function $\varphi(x, n) = \frac{1}{x(1-x)}$ for the decaying resource framework. Then we seek to show that

$$\frac{\partial g}{\partial x} + \frac{\partial}{\partial n}\left[\frac{\epsilon\alpha(x-n)}{x(1-x)}\right] = \frac{\partial g}{\partial x} - \frac{\epsilon\alpha}{x(1-x)} < 0. \tag{49}$$

We are interested in the region of the state space for which limit cycles can be ruled out for any value of ϵ. Notice that the term $-\frac{\epsilon\alpha}{x(1-x)}$ is always negative, but becomes close to zero for small ϵ and x near 1/2. Therefore, limit cycles can be ruled out when $\frac{\partial g}{\partial x} < 0$ for all x, n in the state space. Thus we can rule out cycles for any ϵ when $\delta_H^0 > \delta_L^0$ and $\Delta_L^1 > \Delta_H^1$. We can also rule out cycles when

$$\epsilon > \frac{1}{4\alpha} \max\left(\delta_H^0 - \delta_L^0, \Delta_L^1 - \Delta_H^1\right) \tag{50}$$

holds. This proof of the non-existence of limit cycles shows that limit cycles cannot occur for fast environmental feedbacks. Further, a graphical argument shows that limit cycles cannot occur for slow environmental feedbacks either (given $\Delta_L^1 \delta_H^0 > \delta_L^0 \Delta_H^1$, the fast sub-system is attracting and slow dynamics along the strategy nullcline lead to the equilibrium). Further, when $\Delta_L^1 \delta_H^0 > \delta_L^0 \Delta_H^1$ holds, then we know that the interior equilibrium will be locally stable for all ϵ, which implies that if limit cycles were to arise in this region it would have to be a pair of unstable and stable limit cycles. In extensive simulations we find no evidence of such dynamics. For the renewable resource framework, the same analysis holds, given $e_L > 2e_H - r/q$.

Case 3: ($\Delta_L^1 < 0$ and $\delta_H^0 > 0$) In this case there are either zero or two interior equilibria. In the cases where no interior equilibria are present, limit-cycles cannot occur, since a limit cycle must contain an equilibrium. In the region of the parameter space where there are two interior equilibria, one is always a saddle and the other is stable or unstable. The same analysis as above, in consultation with Figure 2 shows that limit cycles cannot occur in this regime for $\Delta_H^1 < \Delta_L^1$. Further, any limit cycle that does exist will not contain the saddle equilibrium. While this is a proof of the non-existence of limit cycles for only a subset of the green region in Figure 2, we again find no evidence of limit cycles in the green region of the parameter space.

Case 4: ($\Delta_L^1 > 0$ **and** $\delta_H^0 < 0$) Lastly, following similar arguments as in case 3, we conclude that limit cycles cannot occur when $\delta_L^0 < \delta_H^0$.

Supplementary Note 4: Cognitive strategy dynamics as an eco-evolutionary game with decaying resource

Rand et al. [2] present a model of automatic versus controlled processing with a cognition-environment feedback. In their model, the presence of controlled agents leads to an environment that favors automatic processing, and controlled processing is costly. They show that when the cost of control increases when there are fewer control agents, then cycles can result in what they term an "evolutionary pendulum". The minimal model presented in their paper is a special case of our decaying resource framework of game-environment feedbacks. In this section, we make the connection explicit.

First, we introduce the model from Rand et al. [2]. Let x be the fraction of controlled processing agents in the population. Then the fitness of each agent is

$$f_c = 1 - c - w(1-x) \tag{51}$$
$$f_a = 1 - p \tag{52}$$

where c is the fixed cost of controlled processing, w is the extra cost of being a controlled processing agent when rare, and p is the environmentally dependent cost of automatic processing. This cost is governed by a “cognition-environment” feedback given by

$$\dot{p} = \frac{1}{\tau_p}\left((1-x) - p\right) \tag{53}$$

where τ_p governs the relative speed of this feedback. The dynamics of the frequency of the agents is governed by the replicator equation:

$$\dot{x} = x(1-x)(f_c - f_a) \tag{54}$$

This leads a complete system of equations given by

$$\dot{x} = x(1-x)(p - c - w(1-x)) \tag{55}$$
$$\dot{p} = \frac{1}{\tau_p}\left((1-x) - p\right). \tag{56}$$

Recall that the general decaying-resource model presented herein is given by

$$\dot{y} = y(1-y)(\pi_L(y,n) - \pi_H(y,n)) \tag{57}$$
$$\dot{n} = \epsilon\alpha(y - n) \tag{58}$$

where y is the fraction of the population with strategy L, n is a measure of the resource stock and $\pi_L(y,n)$ and $\pi_H(y,n)$ are the environmentally dependent payoffs from the game matrix

$$\Pi(n) = (1-n)\begin{bmatrix} R_0 & S_0 \\ T_0 & P_0 \end{bmatrix} + n\begin{bmatrix} R_1 & S_1 \\ T_1 & P_1. \end{bmatrix} \tag{59}$$

If we transform the equations from Rand et al. [2] by

$$n = p \tag{60a}$$
$$y = 1 - x \tag{60b}$$

we can write the dynamics of the new system as

$$\dot{y} = -\dot{x}(x = 1 - y, p = n) \tag{61}$$
$$\dot{n} = \dot{p}(x = 1 - y, p = n) \tag{62}$$

which simplifies to

$$\dot{y} = y(1 - y)(wy + c - n) \tag{63}$$
$$\dot{n} = \frac{1}{\tau_p}(y - n). \tag{64}$$

Thus, if we let $\epsilon\alpha = 1/\tau_p$, $R_0 = 1$, $S_0 = 1$, $T_0 = 1 - c - w$, $P_0 = 1 - c$, $R_1 = 0$, $S_1 = 0$, $T_1 = 1 - c - w$, and $P_1 = 1 - c$ we can see that our general model decaying resource model (Supplementary Equations 58 and 57) is a linear transformation of the Rand et al. [2] minimal model. Thus the Rand et al. [2] model is a special case of the decaying-resource model presented and analyzed in this paper. Given this, we could typically compute the δ's and from this know the location, stability and dynamics near the equilibria of the system. However, this model is a 'special' special case, thus more analysis is needed to go from the general model to the results of Rand et al. [2].

The δ values that govern the analysis are

$$\Delta_L^1 = T_1 - R_1 = 1 - c - w \tag{65a}$$
$$\Delta_H^1 = P_1 - S_1 = 1 - c \tag{65b}$$
$$\delta_L^0 = R_0 - T_0 = c + w \tag{65c}$$
$$\delta_H^0 = S_0 - P_0 = c \tag{65d}$$

these values for the δ and Δ parameters fall within the special case where

$$\Delta_L^1 - \Delta_H^1 + \delta_L^0 - \delta_H^0 = 0. \tag{66}$$

This is a special case that we have yet to analyze for the general model, because it accounts for a small region of the parameter space. We proceed to analyze the general model given we are in this special case. We are interested in the stability of the interior equilibrium because this is the equilibrium point around which the cyclic dynamics occur in the Rand et al. [2] model.

4.1 Analysis

Assuming that $\Delta_L^1 - \Delta_H^1 + \delta_L^0 - \delta_H^0 = 0$ we analyze the general model with a decaying resource, given by

$$\dot{y} = y(1 - y)g(y, n) \tag{67}$$
$$\dot{n} = \epsilon\alpha(y - n) \tag{68}$$

where y is the fraction of the population with strategy L, n is a measure of the resource stock and

$$g(y,n) = \pi_L(y,n) - \pi_H(y,n) = (1-n)(\delta_L^0 y + \delta_H^0(1-y)) - n(\Delta_L^1 y + \Delta_H^1(1-y)) \tag{69}$$

is the gradient of selection. The interior equilibrium occurs where $g(y,n) = 0$ and $y = n$. The first condition holds when

$$n = \frac{\delta_H^0(1-y) + \delta_L^0 y}{(\Delta_H^1 + \delta_H^0)(1-y) + (\Delta_L^1 + \delta_L^0)y}. \tag{70}$$

In this special case, the denominator is equal to $A = \delta_H^0 + \Delta_H^1 = \delta_L^0 + \Delta_L^1$. This simplifies our analysis and we can write the location of the interior equilibrium as

$$n = 1/A \left[\delta_H^0(1-y) + \delta_L^0 y\right] \tag{71}$$

which implies that

$$n^* = y^* = \frac{\delta_H^0}{\Delta_L^1 + \delta_H^0}. \tag{72}$$

Stability at this interior equilibrium can be derived from the Jacobian matrix evaluated at this equilibrium,

$$J^* = \begin{bmatrix} y(1-y)\frac{\partial g}{\partial y}(y,n) & y(1-y)\frac{\partial g}{\partial n}(y,n) \\ \epsilon\alpha & -\epsilon\alpha \end{bmatrix}_{(y,n)=(y^*,n^*)} \tag{73}$$

where

$$\frac{\partial g}{\partial y}(y^*,n^*) = \frac{\Delta_L^1(\delta_L^0 - \delta_H^0) + \delta_H^0(\Delta_H^1 - \Delta_L^1)}{\delta_H^0 + \Delta_L^1} \tag{74}$$

and

$$\frac{\partial g}{\partial n}(y^*,n^*) = -\delta_H^0 - \Delta_H^1 = -A. \tag{75}$$

An unstable interior equilibrium will occur when $\mathrm{Det}(J^*) > 0$ and $\mathrm{Tr}(J^*) > 0$, and this will lead to cycles. $\mathrm{Det}(J^*) > 0$ if and only if $(\delta_H^0+\Delta_H^1)(\Delta_L^1+\delta_H^0) > \Delta_L^1(\delta_L^0-\delta_H^0)+\delta_H^0(\Delta_H^1-\Delta_L^1)$, which in this special case is equal to

$$\delta_H^0 > -\Delta_L^1 \tag{76}$$

by the definition of A. $\mathrm{Tr}(J^*) > 0$ if and only if $\epsilon\alpha < \frac{\Delta_L^1\delta_H^0\left[\Delta_L^1(\delta_L^0-\delta_H^0)+\delta_H^0(\Delta_H^1-\Delta_L^1)\right]}{(\Delta_L^1+\delta_H^0)^3}$ which in this special case is equal to

$$\epsilon\alpha < \frac{\Delta_L^1\delta_H^0(\Delta_H^1 - \delta_H^0)}{(\Delta_L^1 + \delta_H^0)^2} \tag{77}$$

Now That the analysis has been done for the general case under the assumption that $\Delta_L^1 - \Delta_H^1 + \delta_L^0 - \delta_H^0 = 0$, we can apply it to the model of Rand et al. [2].

4.2 Application to Rand et al. model

In the transformed version of the Rand et al. [2] minimal model we had

$$\Delta_L^1 = 1 - c - w \tag{78}$$
$$\Delta_H^1 = 1 - c \tag{79}$$
$$\delta_L^0 = c + w \tag{80}$$
$$\delta_H^0 = c \tag{81}$$

which can be applied to the conditions for an unstable interior equilibrium in Supplementary Equations 77 and 76. An unstable interior equilibrium occurs when

$$w < 1 \tag{82}$$

and

$$\tau_P > \frac{(1-w)^2}{cw(1-c-w)}. \tag{83}$$

The first condition is satisfied by the assumption needed for the existence of an interior equilibrium, $w + c < 1$ and the assumption that $c \geq 0$ and $w \geq 0$. Notice that when $w = 0$, there does not exist a τ_p for which the interior equilibrium is unstable. This can be seen directly from a necessary condition for cycles, violating Supplementary Condition 24 which requires $w > 0$ (this is from the renewable resource case, but an identical necessary condition exists in the decaying resource case).

Supplementary Note 5: Common-pool resource harvesting

Here, we consider a classic model of common-pool resource harvesting. We assume that individuals either harvest with high, e_H, or low, e_L, effort. We let the evolutionary process be governed by a profit function, $\pi(e_i, \eta)$, that maps the resource level and harvest effort into fitness. As before, we assume that the resource, η is governed by logistic growth, and the harvest rate is proportional to η and effort. We can write our system as

$$\epsilon \dot{x} = x(1-x)\left(\pi(e_L, \eta) - \pi(e_H, \eta)\right) \tag{84}$$
$$\dot{\eta} = r\eta\left(1 - \frac{\eta}{k}\right) - q\eta\left(xe_L + (1-x)e_H\right), \tag{85}$$

where x is the fraction of the population that harvests with low effort, k is the carrying capacity of the resource, q is the efficiency with which effort is transformed into harvest, and ϵ controls the relative timescales of strategy and resource dynamics. For small ϵ, strategy dynamics are fast relative to resource dynamics.

Now, we turn our attention the the profit function, $\pi(e_i, \eta)$. In this simplest case, revenue will depend on the harvest and the price at which the harvested resource can be sold, and costs will increase linearly in effort. Under these assumptions, we can write the profit of an individual as

$$\pi(e_i, \eta) = pq\eta e_i - we_i \tag{86}$$

where p is the fixed price of the resource and w is the marginal cost of effort.

This gives us a complete description of our system, however, it is not immediately obvious that this model is a special case of the renewable resource case studied in detail above. Employing this profit function, we can simply express our system of equations as

$$\epsilon\dot{x} = x(1-x)(e_H - e_L)(w - pq\eta) \tag{87}$$

$$\dot{\eta} = r\eta\left(1 - \frac{\eta}{k}\right) - q\eta\left(xe_L + (1-x)e_H\right). \tag{88}$$

Throughout this analysis, We assume that $e_L < e_H$. Now, in order to make an exact mapping between this model and the general renewable resource model analyzed in Supplementary Note 1 we linearly transform the resource level, η, into an environmental metric, n that is bounded between 0 and 1. Within this transformed space, we can construct a payoff matrix, $\Pi(n)$ that maps onto our common-pool resource harvesting case.

First, we define our environmental metric as

$$n = \frac{\eta - k\left(1 - \frac{qe_h}{r}\right)}{(e_H - e_L)\frac{qk}{r}} \tag{89}$$

and write the state of our resulting system in terms of x and n. We have

$$\epsilon\dot{x} = x(1-x)(e_H - e_L)\left(w + pqk\left(1 - \frac{qe_H}{r}\right) - \frac{pq^2k}{r}(e_H - e_L)n\right) \tag{90}$$

$$\dot{n} = \left(r - q(e_L n + e_H(1-n))\right)(x - n). \tag{91}$$

From this we can reconstruct a payoff matrix $\Pi(n)$ that leads to this system equations. The entries of $\Pi(n)$ are

$$R_0 = S_0 = \left[pqk\left(1 - \frac{qe_H}{r}\right) - w\right]e_L \tag{92}$$

$$T_0 = P_0 = \left[pqk\left(1 - \frac{qe_H}{r}\right) - w\right]e_H \tag{93}$$

$$R_1 = S_1 = \left[pqk\left(1 - \frac{qe_L}{r}\right) - w\right]e_L \tag{94}$$

$$T_1 = P_1 = \left[pqk\left(1 - \frac{qe_L}{r}\right) - w\right]e_H. \tag{95}$$

Thus, we can write the values of δ as

$$\Delta_L^1 = \Delta_H^1 = \left[pqk\left(1 - \frac{qe_L}{r}\right) - w\right](e_H - e_L) \tag{96}$$

$$\delta_H^0 = \delta_L^0 = -\left[pqk\left(1 - \frac{qe_H}{r}\right) - w\right](e_H - e_L). \tag{97}$$

Depending on the parameter values chosen we have three possible outcomes. First, when positive profits occur at the low effort dominated environmental equilibrium but negative profits result at the high impact dominated equilibrium then we have $\Delta_L^1 = \Delta_H^1 > 0$ and $\delta_H^0 = \delta_L^0 > 0$. This implies that the system always falls at the boundary of the region where cycles are possible in Figure 1b. This implies that while cycles cannot occur in this system,

small changes to the payoff structure could lead to cycles. In particular, holding all else equal, increasing Δ_H^1 or δ_L^0 would permit cycles.

Second, if e_H is low enough that $pqk\left(1 - \frac{qe_H}{r}\right) - w > 0$, then $\delta_H^0 = \delta_L^0 < 0$ and $\Delta_H^1 = \Delta_L^1 > 0$. Thus, by referencing Figure 1d, we conclude that the only stable outcome is the high-impact equilibrium. Third, if both $pqk\left(1 - \frac{qe_H}{r}\right) - w < 0$ and $pqk\left(1 - \frac{qe_L}{r}\right) - w < 0$, then we have $\delta_H^0 = \delta_L^0 > 0$ and $\Delta_L^1 = \Delta_H^1 < 0$. As illustrated in Figure 1c, this implies that the low impact dominated state will be the equilibrium outcome. We assumed ex-ante that $e_L < e_H$ and thus these are the only possible outcomes within this system.

Supplementary Note 6: Market pricing CPR model

While the simplest common-pool resource harvesting model cannot produce cyclic dynamics, one possible driver could be market pricing. Whereas in the previous section the price received for each unit of harvested resource was constant, here we let the price received be a function of the total harvest at the present time, to mimic market pricing. A fixed price makes sense in the case where the system in question represents a small part of a large or global market because then local supply can have only a small impact on the global market price. Here, we allow harvest quantity to effect the price. This is more applicable to cases where the markets for the resource are local or the suppliers in consideration account for a significant portion of total supply. As a first approximation, we assume that the market price is linearly decreasing in the quantity supplied.

This alters that profit function, resulting in

$$\pi(e_i, x, n) = p(h)qne_i - we_i \tag{98}$$

where $h = nq(xe_L + (1-x)e_H)$ and $p(h)$ is the market price as a function of resource supply. This model is not a special case of the model presented in Supplementary Note 1 since there are higher order terms in the dynamical equations introduced through the dependency of the price of the resource on the state of the system. We consider the linear price equation,

$$p(h) = p_0(1 - \gamma h) \tag{99}$$

so that there is a price ceiling of p_0 and a linear decrease with slope γ as supply increases. With this formulation, we can write the profit of low and high effort harvesters as

$$\pi_L(x, n) = p_0(1 - \gamma nq(xe_L + (1-x)e_H))qne_L - we_L \tag{100}$$

$$\pi_H(x, n) = p_0(1 - \gamma nq(xe_L + (1-x)e_H))qne_H - we_H. \tag{101}$$

In total, the model can be written as

$$\epsilon\dot{x} = x(1-x)\left(\pi_L(x,n) - \pi_H(x,n)\right) \tag{102}$$

$$\dot{n} = rn\left(1 - \frac{n}{k}\right) - qn\left(xe_L + (1-x)e_H\right). \tag{103}$$

Bifurcation analysis allows us to show how equilibria (and their stability) change as a function of key parameters of interest. First, we examine the effect of the slope of the price

function on the equilibrium fraction of low-effort harvesters. this slope, γ, is a measure of how quickly price declines as supply increases and is related to the price elasticity. The equilibrium state of the resource can also be displayed, however, in our model there is a linear relationship between the fraction of low-effort harvesters and the equilibrium state of the resource. Thus, we only present the bifurcation diagram in the fraction of low-effort harvesters, x. Supplementary Figure 1a shows that under regime with a high maximum price, p_0, increasing the rate at which harvest quantity depresses market price, γ, leads to hysteresis and multiple stable states. On the other hand, Supplementary Figure 1b shows that under a low maximum price, increasing γ leads to a smooth transition from one equilibrium to the other, without a region of multiple stable states.

6.1 Market pricing analysis

The interior strategy nullcline of the system changes from previous models because market pricing of harvest changes the set of points where zero profits result. Letting $\pi(e_L, x, n) - \pi(e_H, x, n) = 0$ and solving for n gives

$$n = \frac{p_0 \pm \sqrt{p_0(p_0 - 4\gamma w(xe_L + (1-x)e_H)}}{2p_0\gamma q(xe_L + (1-x)e_H)} \tag{104}$$

so that there are two branches of the relationship depending on whether the square root is added or subtracted from the relationship. This can be simplified to

$$n = \frac{2w}{p_0 q \pm q\sqrt{p_0}\sqrt{p_0 - 4\gamma w(e_H(1-x) + e_L x)}} \tag{105}$$

which, since $e_H > e_L$, illustrates that as x increases, the second term in the denominator also increases. This implies that on the upper branch of the nullcline is increasing in x and the lower branch of the nullcline is decreasing in x for all $x \in [0, 1]$, given that $p_0 > 4\gamma w(e_H(1-x) + e_L x)$. This will simplify stability analyses at interior equilibria.

Now we characterize the stability of the equilibria of the system. First, we will consider the interior equilibria.

Interior equilibria At interior equilibria, the environmental nullcline intersects with the strategy nullcline. The analysis will depend on the relative slopes of these nullclines, and whether the intersection occurs at the upper or lower branch of the strategy nullcline. Consider the Jacobian matrix

$$J = \begin{bmatrix} \frac{\partial \dot{x}}{\partial x} & \frac{\partial \dot{x}}{\partial n} \\ \frac{\partial \dot{n}}{\partial x} & \frac{\partial \dot{n}}{\partial n} \end{bmatrix}_{(x^*, n^*)} \tag{106}$$

at the lower branch of the strategy nullcline. We know that the environment nullcline is increasing in x because $e_L < e_H$, and that $\dot{n} > 0$ below the nullcline. Further, from the form of the strategy nullcline, we know that the lower branch is decreasing in x and that $\dot{x} > 0$ below the nullcline. From this we can determine the sign of each element of the Jacobian matrix. In this case we have

$$J = \begin{bmatrix} - & - \\ + & - \end{bmatrix} \tag{107}$$

for the signs of the Jacobian, which implies stability.

Along the upper branch of the nullcline, the stability analysis is slightly more complex. Consider the path derivatives along the $\dot{x} = 0$ and $\dot{n} = 0$ nullclines. By construction, the value of the path derivative is equal to zero, thus we can write

$$\frac{\partial \dot{x}}{\partial n}\frac{\partial n}{\partial x} + \frac{\partial \dot{x}}{\partial x} = 0 \tag{108}$$

for the path derivative along the strategy nullcline where the slope of the nullcline is $\frac{\partial n}{\partial x}$. Similarly, we can write the value of the path derivative along the environment nullcline as

$$\frac{\partial \dot{n}}{\partial n}\frac{\partial n}{\partial x} + \frac{\partial \dot{n}}{\partial x} = 0. \tag{109}$$

Let S_x and S_n denote the slopes of the strategy and environment nullclines at equilibrium, respectively. Using these identities, we can rewrite our Jacobian as

$$J = \begin{bmatrix} -\frac{\partial \dot{x}}{\partial n} S_x & \frac{\partial \dot{x}}{\partial n} \\ -\frac{\partial \dot{n}}{\partial n} S_n & \frac{\partial \dot{n}}{\partial n} \end{bmatrix}_{(x^*, n^*)} \tag{110}$$

since S_n is positive and $\dot{n} > 0$ below the environment nullcline then $\frac{\partial \dot{n}}{\partial n}(x^*, n^*) < 0$. Also, since S_x is positive and $\dot{x} > 0$ above the upper branch of the strategy nullcline, then we can conclude that $\frac{\partial \dot{x}}{\partial n}(x^*, n^*) > 0$. In total, this implies that $\mathrm{Tr}(J) < 0$ and

$$\mathrm{Det}(J) = \frac{\partial \dot{n}}{\partial n}\frac{\partial \dot{x}}{\partial n}\left(S_n - S_x\right). \tag{111}$$

We can conclude that the interior equilibrium at an intersection of the upper branch of the strategy nullcline with the environment nullcline will be stable if and only if $S_x > S_n$. If the slope of the environment nullcline is greater than the strategy nullcline, then the interior equilibrium at this point will be a saddle. Note that never does the stability of an interior equilibrium depend on the relative timescale of resource and strategy dynamics, ϵ.

Edge equilibria Here, we analyze the stability of the equilibria at the edge of the phase space. These analyses are simplest graphically. Given that $e_L < e_H$ and e_L is low enough that it will not drive the environment to zero, then both equilibria at $n = 0$ will be unstable. The equilibrium at $x = 0$ and $n > 0$ is stable if and only if it's location falls above the lower branch of the strategy nullcline, but below the upper branch of the strategy nullcline. Conversely, the equilibrium at $x = 0$ and $n > 0$ is stable if and only if its location falls below the lower, or above the upper branch of the strategy nullcline.

Supplementary Figure 2 shows a panel of eight qualitatively distinct phase diagrams of our model of market pricing, harvest and environmental dynamics. While there are many possible qualitative outcomes, none involve cyclic dynamics.

Supplementary Note 7: Frequency dependent harvesting efficiency CPR model

In this section we extend the common-pool resource model introduced in Supplementary Note 4 with the addition of a term to account for the dependency of harvest efficiency, q,

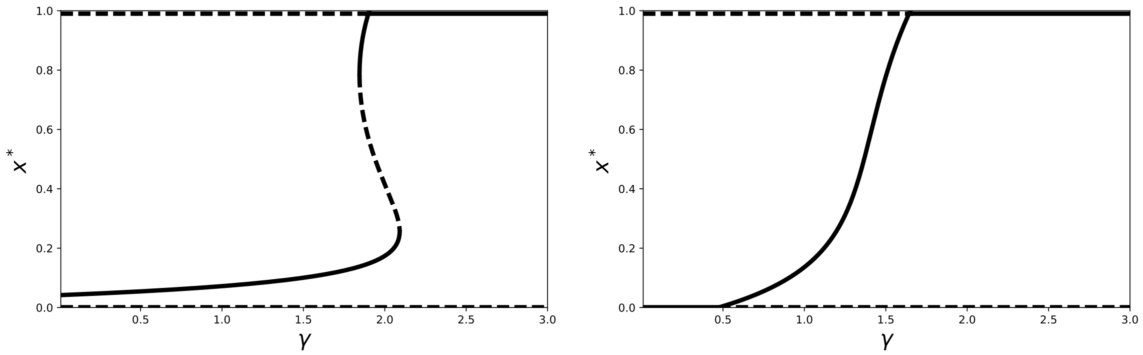

(a) High p_0 (b) Low p_0

Supplementary Figure 1: Bifurcation diagrams of equilibria in the market pricing model illustrating stable (solid line) and unstable (dashed line) equilibrium frequencies of low-effort harvesting under high and low price regimes showing multiple equilibria and hysteresis in the downward slope of the market price, γ, under a high p_0 and a smooth transition from high-effort dominance to low effort dominance under a low p_0 regime.

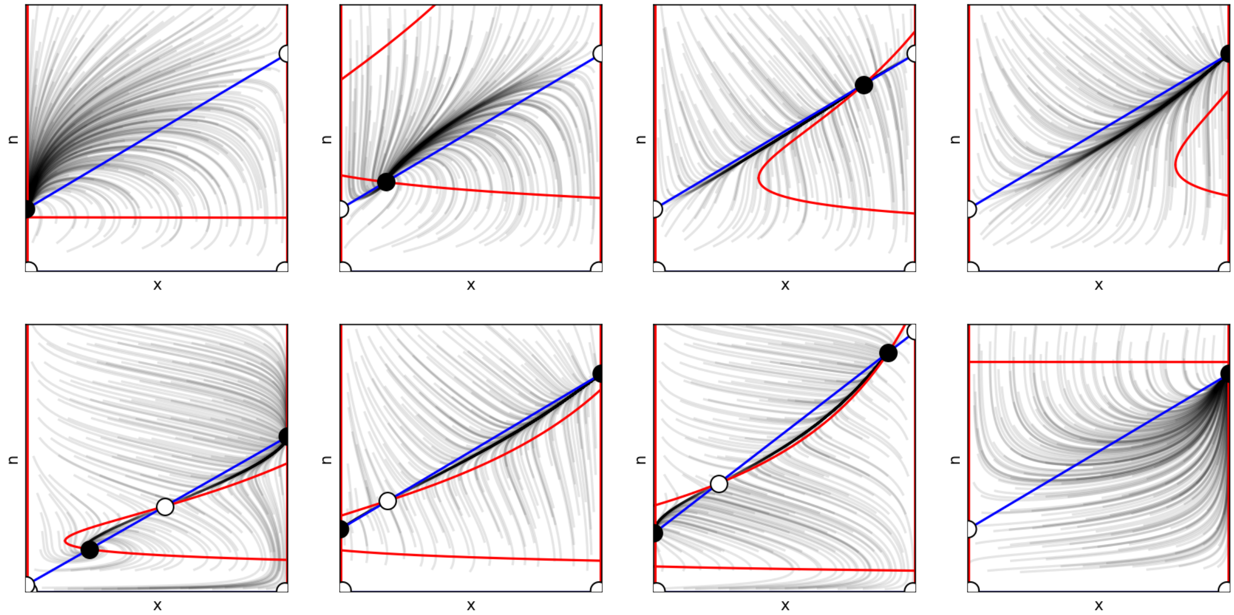

Supplementary Figure 2: Phase planes under eight qualitatively distinct scenarios that can arise under market pricing. Red curves are strategy nullclines, where $\dot{x} = 0$. Blue lines are environment nullclines, where $\dot{n} = 0$. Equilibria are marked with circles, and occur at intersections of the strategy and environment nullclines. Open circles correspond to unstable equilibria, and closed circles correspond to stable equilibria.

parameter	1(a)	1(b)	2(a)	2(b)	2(c)	2(d)	2(e)	2(f)	2(g)	2(h)
r	0.3	0.3	0.3	0.3	0.3	0.3	0.36	0.3	0.36	0.3
k	6	6	4	4	4	4	39.6	4	39.6	4
q	0.5	0.5	0.5	0.5	0.5	0.5	0.59	0.5	0.59	0.5
e_L	0.276	0.253	0.25	0.25	0.25	0.25	0.357	0.25	0.185	0.25
e_H	0.564	0.49	0.5	0.5	0.5	0.5	0.6	0.5	0.515	0.5
p_0	50	25	35	35	40	35	13.7	35	13.7	35
w	12	12	10	12	10	10	21.7	5	16.6	43
ϵ	20	20	20	20	20	20	20	20	20	20
γ	0-3	0-3	0.1	1.3	2.5	2.9	0.28	3.2	0.28	0.001

Supplementary Table 1: Parameter values used to create each phase plane shown in Supplementary Figure 1 and Supplementary Figure 2, moving left to right, from top-left (a) to bottom-right (h)

on the frequency of a strategy. This model is not a special case of the general renewable resource model outlined in Supplementary Note 1 because the frequency dependent harvest efficiency terms alter the dynamics of the resource. The payoff function, however, is a special case of the payoff matrices considered in Supplementary Note 1. Suppose that low effort harvesters share information about the state of and location of the resource. This could lead to increasing harvest efficiency, q, as a function of the fraction of the population who harvest with low effort, x. Alternatively, each strategy may require specialized skills and labor. As a strategy increases in frequency, the supply of these skills increases, potentially leading to efficiency gains. In Supplementary Figure 3 dynamics are shown for the case where low effort harvesters have greater efficiency increases in their frequency. This can lead to limit cycles depending on the relative speed of the resource dynamics. We formalize this by considering a catchability function, $q(x)$ that increases linearly in x for low effort harvesters, and decreases in x for high effort harvesters. This indicates that when a particular strategy is more common, the harvest of the resource by players with that strategy becomes more efficient. This could result form communication about the location of the resource, which would lead to an increase in harvest per unit effort for a given environmental state.

Our model of a common-pool resource with variable harvesting efficiency is analogous to models of ostracism in common-pool systems [3, 4]. While the details differ, both models feature added costs associated with strategies that are at low frequency – and the dynamical implications are qualitatively the same. As this analogy illustrates, there are many causes of frequency dependence in common-pool resource systems, and the framework we have developed allows the consequences to be understood generally.

Specifically, we extend the basic model of Supplementary Note 5 by incorporating frequency dependent catchability. For low effort harvesters, we let $q_L(x) = q_0(1 + \alpha_L x)$ and for high effort harvesters we let $q_H(x) = q_0(1 + \alpha_H(1 - x))$ such that, all else being equal, harvest rates increase with increase prevalence of a strategy, and the magnitude of the benefit of increased frequency is controlled by α_L and α_H. The profit of each strategy can be

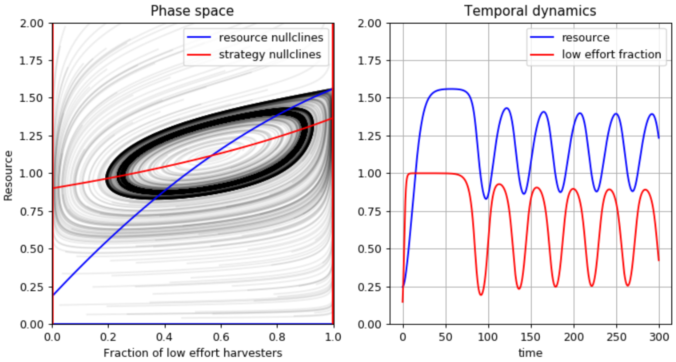

Supplementary Figure 3: Phase plane and temporal dynamics of common-pool resource harvesting with frequency-dependent harvesting efficiency. The dynamics show convergence to a stable limit cycle. $(r = 0.33, K = 4, q = 0.5, e_L = 0.33, e_H = 0.6, p = 10, w = 5, \epsilon = 1.3, \alpha_L = 0.22, \alpha_H = 0.05)$

written as

$$\pi_L(x,n) = pq_0(1+\alpha_L x)ne_L - we_L \tag{112}$$

$$\pi_H(x,n) = pq_0(1+\alpha_H(1-x))ne_H - we_H \tag{113}$$

With these modifications, our system can be written as

$$x' = x(1-x)\left(pnq_0(e_L(1+\alpha_L x) - e_H(1+\alpha_H(1-x))) - w(e_L - e_H)\right) \tag{114}$$

$$n' = \epsilon rn\left(1-\frac{n}{k}\right) - \epsilon q_0 n\left(e_L x(1+\alpha_L x) + e_H(1-x)(1+\alpha_H(1-x))\right), \tag{115}$$

given that time has been re-scaled.

To analyze this system we consider the nullclines. The sets of points where $n' = 0$ are $n = 0$ and

$$n = k\left(1 - \frac{q_0}{r}(e_L x(1+\alpha_L x) + e_H(1-x)(1+\alpha_H(1-x)))\right). \tag{116}$$

The sets of points where $x' = 0$ are $x = 0$, $x = 1$ and

$$n = \frac{w(e_H - e_L)}{pq_0\left[(e_H + e_H\alpha_H - e_L) - (e_H\alpha_H + e_L\alpha_L)x\right]}. \tag{117}$$

Since $e_H > e_L$ we know that the strategy nullcline is increasing in x when the strategy nullcline lies within the state space, $n > 0$. Further, the resource nullcline is quadratic in x and opens downward. We are in particular interested in the stability of interior equilibria. Stability at interior equilibria depends on the relative timescale of resource and strategy dynamics, as well as on the slopes of the nullclines. Consider the path derivatives of the resource and strategy dynamics equations along their respective nullclines, which are equal to zero, by construction. This allows us to write the Jacobian Matrix as

$$J = \begin{bmatrix} -\frac{\partial x'}{\partial n}S_x & \frac{\partial x'}{\partial n} \\ -\frac{\partial n'}{\partial n}S_n & \frac{\partial n'}{\partial n} \end{bmatrix}_{(x^*,n^*)}. \tag{118}$$

Stability at the equilibrium can be determined from the trace and determinant of J. We have

$$\mathrm{Det}(J) = \frac{\partial n'}{\partial n}\frac{\partial x'}{\partial n}\left(S_n - S_x\right). \tag{119}$$

The structure of our system guarantees that $\frac{\partial n'}{\partial n}$ and $\frac{\partial x'}{\partial n}$ will be negative at equilibrium. Therefore, $S_n > S_x$ is necessary, but not sufficient for stability. If $S_x > S_n$ the interior equilibrium will be a saddle. Given that $S_n > S_x$, then stability will occur when when the trace of J is negative. Due to our separation of timescales, we have a term, ϵ, that controls the relative speed of resource dynamics. We have

$$\mathrm{Tr}(J) = \frac{\partial n'}{\partial n} + \frac{\partial x'}{\partial x} \tag{120}$$

with $\frac{\partial n'}{\partial n} < 0$ and $\frac{\partial x'}{\partial x} > 0$. Since the first term contains ϵ, we know that the trace will be negative, and stability will result for sufficiently fast resource dynamics (large ϵ) and conversely the equilibrium will be unstable for slow resource dynamics.

While we can solve for the critical value of ϵ that separates parameter regions leading to stability with those leading to an unstable equilibrium and a limit-cycle, the closed form solution is unwieldy and does not enhance understanding. However, we can use this expression to quickly find the critical value of ϵ for any set of parameter values.

Changes in r, the intrinsic rate of growth of the resource, not only change the speed of resource dynamics, but also the location and stability of the equilibria of the system. For low r, corresponding to resources with low productivity, only low effort harvesting is stable. As r increases high effort harvesters can be supported at equilibrium, but this equilibrium is in close proximity to the low effort dominated equilibrium which is no longer stable, but only just so. Intuitively, this makes limit cycles less likely to occur because dynamics pass near two equilibria, slowing strategy dynamics and limiting environmental overshoot that drives cyclic dynamics. As a result, limit cycles occur for larger values of ϵ when the interior equilibrium is near the center of the state space. This highlights the non-monotonicity of the critical value of ϵ in r. For a fixed value of ϵ, increasing r can destabilize, then stabilize the system (see Supplementary Figure 4).

As α_L and α_H get larger, the strength of positive frequency dependence increases. This can lead to sets of nullclines that cross twice in the interior of the state space. At the intersection where the resource nullcline has a greater slope than the strategy nullcline stability depends on ϵ, as before. The other interior equilibrium will be a saddle, and one of the edge equilibria will be stable. This can lead to three outcomes. First, if both the interior and edge equilibrium are stable, then there will be dependence on initial conditions with dynamics either leading to the interior or edge equilibrium. Next, if ϵ is small, the interior equilibrium becomes unstable and a limit cycle forms around it due to a Hopf bifurcation. In this case there is still dependence on initial conditions where either a limit cycle or an edge equilibrium result. Finally, if ϵ is too small, the interior equilibrium is unstable and no limit cycle results, all initial conditions lead to the edge equilibrium.

To illustrate this complexity, we consider a range of cases with where only r changes. When we let $\epsilon = 2.1$, increasing r can first de-stabilize the interior equilibrium, then stabilize it. This highlights the non-monotonicity of the critical value of ϵ in r. Supplementary Figure 4 shows that intermediate values of r can lead to cyclic dynamics. This is in contrast

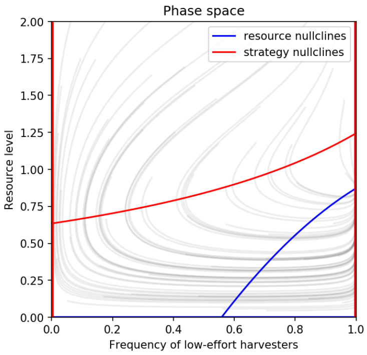

(a) Dynamics for $r = .25$

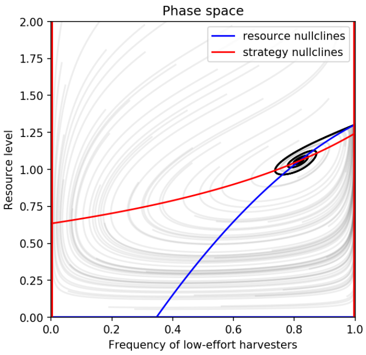

(b) Dynamics for $r = .29$

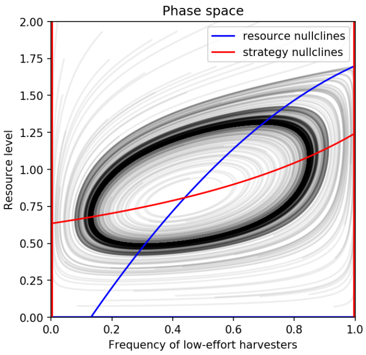

(c) Dynamics for $r = .34$

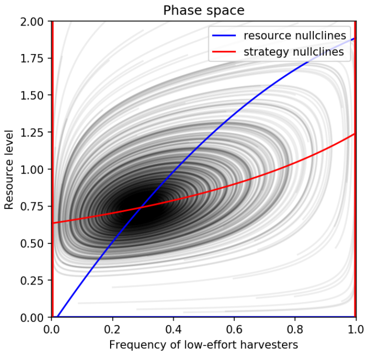

(d) Dynamics for $r = .37$

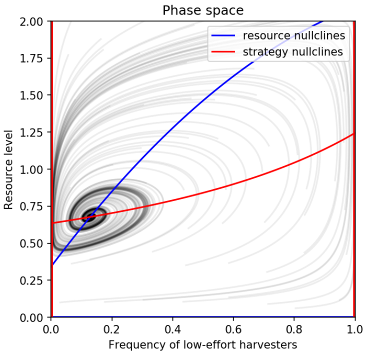

(e) Dynamics for $r = .41$

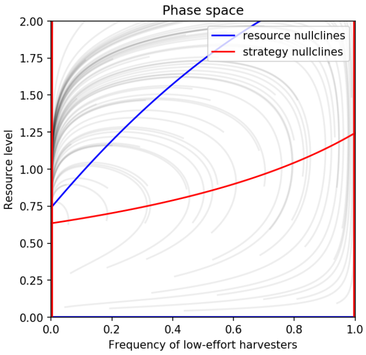

(f) Dynamics for $r = .46$

Supplementary Figure 4: As r, the intrinsic rate of growth of the resource, increases, the speed of resource dynamics increase and the location of the equilibria change. As opposed to changes in ϵ where higher values always stabilize an interior equilibrium, increasing r can either stabilize or de-stabilize an interior equilibrium depending on context. Here we show that all else being equal, increase r can lead from stable equilibria, to cycles and back. ($\epsilon = .25, q = .5, K = 4, p = 10, w = 5, e_L = .34, e_H = .6, \alpha_L = .15, \alpha_H = .25$)

to the stability condition under the renewable resource model where we found that both r and ϵ had similar effects of stability.

Supplementary Note 8: Environmental feedback with tipping points

Here we consider models in which two environmental extremes can be reached, dependent on the strategy profile of the population. We broadly categorize these models as describing environments with tipping points, because there are critical thresholds in strategy frequencies at which environmental dynamics tend toward one (or the other) extreme environment. Intermediate environmental states can be considered as either the fraction of the environment that is in each alternate state or as the environmental metric that describe the entire environment.

In contrast to the renewing and decaying environmental feedbacks studies in the rest of this paper, when we model environmental feedback with tipping points, the dynamical equations for the environment all have $n(1-n)$ terms. These terms create two new edge equilibria at $(x^*, n^*) = (1, 0)$ and $(x^*, n^*) = (0, 1)$. These equilibria will always be either unstable nodes or unstable saddle equilibria depending on whether the incentive to be a follower of strategy change is positive or negative at these corners.

Our analysis of environmental feedback with tipping points focuses on how these types of feedbacks alter the stability criteria for an internal equilibrium that has the potential to be either stable or unstable. However, when environmental feedbacks arise via tipping points, the existence criteria for, and location of, interior equilibria change relative to the cases of renewing and decaying environmental feedbacks. We beg the question when and where interior equilibria will arise, and instead focus on the stability of such an interior equilibrium, given that one exists.

8.1 Model of Weitz et al.

Weitz et al. [1] study an environmental feedback where the environment responds directly to the strategies that individuals employ.

Weitz et al. [1] consider the model

$$\dot{x} = x(1-x)g(x,n) \tag{121}$$
$$\dot{n} = \tilde{\epsilon}n(1-n)((1+\theta)x-1) \tag{122}$$

where $g(x,n) = \pi_L - \pi_H$ is the incentive to switch to strategy 1 given the current state of the environment and strategy mix of the population, and $\frac{1}{1+\theta}$ is the critical value of x at which the direction of dynamics of n switches.

Weitz et al. [1] identify an oscillatory tragedy of the commons as resulting from an unstable interior equilibrium of the system. The stability criteria of the system at such a state can be understood in terms of the Jacobian matrix

$$J^* = \begin{bmatrix} x(1-x)\frac{\partial g}{\partial x}(x,n) & x(1-x)\frac{\partial g}{\partial n}(x,n) \\ \tilde{\epsilon}n(1-n)(1+\theta) & 0 \end{bmatrix}_{(x,n)=(x^*,n^*)} \tag{123}$$

Trace-determinant analysis implies that an interior equilibrium will be unstable when

$$\frac{\partial g}{\partial x}(x^*, n^*) > 0 \tag{124}$$

and

$$\frac{\partial g}{\partial n}(x^*, n^*) < 0 \tag{125}$$

where

$$\frac{\partial g}{\partial n}(x^*, n^*) = -\left[(\Delta_H^1 + \delta_H^0)(1-x^*) + (\delta_L^0 + \Delta_L^1)x^*\right] \tag{126}$$
$$\frac{\partial g}{\partial x}(x^*, n^*) = (\delta_L^0 - \delta_H^0)(1-n^*) + (\Delta_H^1 - \Delta_L^1)n^* \tag{127}$$

and

$$(x^*, n^*) = \left(\frac{1}{1+\theta}, \frac{\delta_H^0\theta + \delta_L^0}{(\Delta_H^1 + \delta_H^0)\theta + \Delta_L^1 + \delta_L^0}\right). \tag{128}$$

In the model of Weitz et al. [1] the stability criteria do not depend on the relative timescale of strategy and environmental dynamics because the environmental nullcline is vertical at the interior equilibrium. This is the reason that the Jacobian matrix has an entry equal to zero, and hence there is no $\tilde{\epsilon}$ term in the equation for the trace.

8.2 Simple tipping point model

Now we consider a simpler model that nonetheless captures the regimes found in Weitz et al. [1].

Let the system be governed by

$$\dot{x} = x(1-x)g(x,n) \tag{129}$$

$$\dot{n} = \epsilon n(1-n)(x-\mu) \tag{130}$$

where μ is a fixed threshold in x at which environmental dynamics switch directions.

The Jacobian matrix at an interior equilibrium for this model is

$$J^* = \begin{bmatrix} x(1-x)\frac{\partial g}{\partial x}(x,n) & x(1-x)\frac{\partial g}{\partial n}(x,n) \\ \epsilon n(1-n) & 0 \end{bmatrix}_{(x,n)=(x^*,n^*)} \tag{131}$$

Again, trace-determinant analysis implies that an interior equilibrium will be unstable when

$$\frac{\partial g}{\partial x}(x^*, n^*) > 0 \tag{132}$$

and

$$\frac{\partial g}{\partial n}(x^*, n^*) < 0 \tag{133}$$

In this model the interior equilibrium has the form

$$(x^*, n^*) = \left(\mu, \frac{\delta_H^0(1-\mu) + \delta_L^0\mu}{(\Delta_H^1 + \delta_H^0)(1-\mu) + (\Delta_L^1 + \delta_L^0)\mu}\right). \tag{134}$$

When $\mu = 1/1+\theta$ and time is re-scaled by letting $\epsilon = \tilde{\epsilon}(1+\theta)$, then this simple model is the same as the Weitz et al. [1] model.

8.3 Environments with a distribution of tipping points

Now we consider a model that generates non-linear nullclines. Suppose that the critical thresholds for environmental states to shift are governed by a distribution. For example suppose that strategy thresholds for environmental tipping points are truncated normally distributed in x, i.e. $x \sim N(\mu, \sigma, 0, 1)$. Other distributions will also work for this model, and if the domain of the distribution is $[0, 1]$, then the environmental nullcline will pass through the corners of the phase plane, as in the models of renewing and decaying resources that we

have analyzed. Let $F(x)$ be the cumulative distribution of x. Then we can write our system as

$$\dot{x} = x(1-x)g(x,n) \tag{135}$$
$$\dot{n} = \epsilon n(1-n)(x - F^{-1}(n)) \tag{136}$$

where F^{-1} is the inverse CDF. This model converges to the model of Weitz et al. [1] as $\sigma \to 0$, $\mu = 1/(1+\theta)$ and $\epsilon = \tilde{\epsilon}(1+\theta)$, and resembles our models of renewing and decaying resource linkages as $\sigma \to \infty$.

The Jacobian of this system is

$$J^* = \begin{bmatrix} x(1-x)\frac{\partial g}{\partial x}(x,n) & x(1-x)\frac{\partial g}{\partial n}(x,n) \\ \epsilon n(1-n) & -\epsilon n(1-n)\frac{\partial F^{-1}}{\partial n}(n) \end{bmatrix}_{(x,n)=(x^*,n^*)} \tag{137}$$

Analysis of this system can be simplified by considering the path derivatives of each component of the system along the its nullclines. First for the $\dot{x}$ equation we have

$$0 = \frac{\partial \dot{x}}{\partial x} + \frac{\partial \dot{x}}{\partial n} S_x \tag{138}$$

where $S_x = \frac{\partial n}{\partial x}$ is the slope of the $\dot{x}$ nullcline defined by $g(x,n) = 0$. We know that $\frac{\partial \dot{x}}{\partial x} + \frac{\partial \dot{x}}{\partial n} S_x$ is equal to zero because it is the path derivative of $\dot{x}$ along a nullcline where $\dot{x} = 0$.

Next, along the interior nullcline of the $\dot{n}$ equation, given by $n = F(x)$, we have

$$0 = \frac{\partial \dot{n}}{\partial x} + \frac{\partial \dot{n}}{\partial n} S_n \tag{139}$$

where $S_n = \frac{\partial n}{\partial x}$ is the slope of the interior $\dot{n}$ nullcline. Also, note that from the expression for the nullcline, we know that

$$S_n = \frac{\partial F}{\partial x}, \tag{140}$$

and by extension, we have

$$\frac{\partial F^{-1}}{\partial n} = \frac{1}{S_n}. \tag{141}$$

These path derivatives allow us to rewrite the determinant as

$$\text{Det}(J^*) = \frac{\partial \dot{x}}{\partial x}\frac{\partial \dot{n}}{\partial n} - \frac{\partial \dot{x}}{\partial n}\frac{\partial \dot{n}}{\partial x} = \frac{\partial \dot{x}}{\partial n}\frac{\partial \dot{n}}{\partial n}(S_n - S_x) \tag{142}$$

So that we have a positive determinant when

$$-\epsilon x^* n^*(1-x^*)(1-n^*)\frac{\partial g}{\partial n}(x^*,n^*)\left(1 - \frac{S_x}{S_n}\right) > 0 \tag{143}$$

which occurs when $\frac{\partial g}{\partial n}(x^*,n^*) < 0$ and $S_n > S_x$.

Further, the trace of the Jacobian will be positive, $\text{Tr}(J^*) > 0$ (which along with a positive determinant implies an unstable equilibrium) when

$$\epsilon n^*(1-n^*)\frac{1}{S_n} < x^*(1-x^*)\frac{\partial g}{\partial x}(x^*,n^*). \tag{144}$$

Relation to Weitz et al. [1] The criteria for an unstable interior equilibrium in this model are different from those under the model studied by Weitz et al. [1]. However, when the variance of the distribution goes to zero, and it's mean goes to $\frac{1}{1+\theta}$ we have an exact correspondence between the stability criteria of the two models. Critically, as the variance of the distribution decreases, the slope the the interior nullcline increases. Thus as $\sigma \to 0$ and this model approaches that of Weitz et al. [1], because $S_n \to \infty$. In this limit, the condition for a positive determinant becomes

$$\frac{\partial g}{\partial n}(x^*, n^*) < 0, \tag{145}$$

and the condition for a positive trace becomes

$$\frac{\partial g}{\partial x}(x^*, n^*) > 0. \tag{146}$$

In this limit, our model reproduces the result of Weitz et al. [1] that states that stability of the interior equilibrium does not depend on ϵ. However, this is only the case as $S_n \to \infty$. For a finite value of S_n (as occur in our model for $\sigma \neq 0$) a sufficiently large value for ϵ, implying fast environmental dynamics, will stabilize an interior equilibrium.

That stability of an interior equilibrium depends on ϵ is a general feature found in both renewing, decaying, and threshold environmental response models for all but the limiting case where $\sigma = 0$.

8.4 Linear case

Next, we consider a linear implementation of the previous model, which occurs when the distribution of environmental tipping points is uniform. We assume that there is a uniform distribution in x of width a, centered at μ. Now, we replace the μ term in the previous model with the inverse of the CDF of the uniform distribution of thresholds. This leads to a system governed by

$$\dot{x} = x(1-x)g(x, n) \tag{147}$$
$$\dot{n} = \epsilon n(1-n)(x - an + a/2 - \mu). \tag{148}$$

The Jacobian of this system is

$$J^* = \begin{bmatrix} x(1-x)\frac{\partial g}{\partial x}(x, n) & x(1-x)\frac{\partial g}{\partial n}(x, n) \\ \epsilon n(1-n) & -\epsilon n(1-n)a \end{bmatrix}_{(x,n)=(x^*,n^*)} \tag{149}$$

which will have a positive determinant when

$$a\frac{\partial g}{\partial x}(x^*, n^*) + \frac{\partial g}{\partial n}(x^*, n^*) < 0 \tag{150}$$

and a positive trace (implying an unstable fixed point) when

$$\epsilon n^*(1-n^*)a > x^*(1-x^*)\frac{\partial g}{\partial x}(x^*, n^*). \tag{151}$$

First, two things to note about these criteria for an unstable interior equilibrium:

1. When $a = 0$, then the condition exactly aligns with the previously considered models in Supplementary Notes 8.2 and 8.1.

2. When $\mu = 1/2$ and $a = 1$, then the stability criterion for the interior equilibrium closely matches those found in both the renewable and decaying resource models considered in our paper, with only the addition of the $n(1-n)$ terms differing between the models.

In general, the only case in which ϵ does not come into the criteria for an oscillating dynamic is the limiting case where $a = 0$. Mathematically, this is because of the final entry of the Jacobian matrix becomes zero when $a = 0$. Graphically, this can be seen because the environmental nullcline becomes vertical. As we slightly relax the system away from this limiting case (where $a = 0$) we see that timescales do indeed matter, with fast environmental feedbacks having a stabilizing effect.

parameter	5(a)	5(b)	5(c)	5(d)	5(e)	5(f)
ϵ	1	1	2	2	1/2	1/2
μ	–	1/2	1/2	1/2	1/2	1/2
θ	1	–	–	–	–	–
σ	–	–	–	1/10	–	1/10
a	–	–	3/10	–	3/10	–
Δ_L^1	2	2	2	2	2	2
Δ_H^1	1	1	1	1	1	1
δ_L^0	3	3	3	3	3	3
δ_H^0	1	1	1	1	1	1

Supplementary Table 2: Parameter values used to generate each panel in Supplementary Figure 5

Supplementary Note 9: Relationship to Sigdel et al.

Sigdel et al. [5] present a model of forest growth and opinion dynamics. Their model illustrates the role that social norms have on the dynamics of opinions, and thus forest cover. Their model is of the form

$$\dot{x} = \kappa x(1-x)\left[c - F + \xi(2x-1)\right] \tag{152}$$

$$\dot{F} = RF(1-F) - h(1-x)F \tag{153}$$

where x is the fraction of the population with a pro-conservation opinion, F is the degree of forest cover, c is the conservation value of forest, κ is the social learning rate, ξ is the strength of social norms, R is the regeneration rate of forest, and h is the harvest rate.

In the case that $h < R$ the environment approaches the state $F = 1$ when the population strategy is fixed at $x = 1$. When the strategy profile of the population is fixed at $x = 0$, then the environment approaches $F = 1 - h/R > 0$. In this case, where $h < R$, the model of Sigdel et al. [5] is a special case of the renewing resource framework that we analyze. The

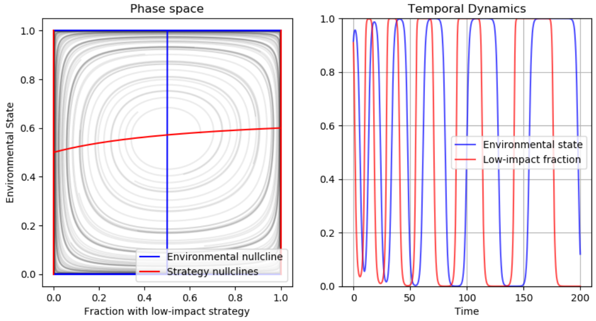

(a) Weitz et al. [1] model

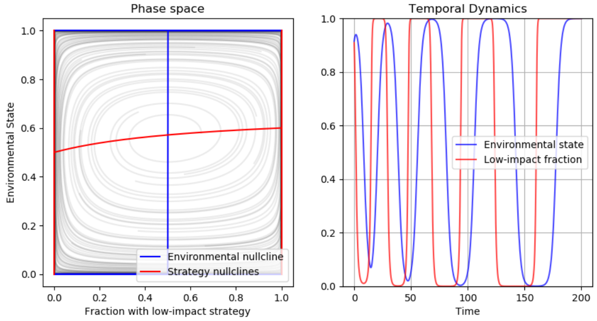

(b) Simple model

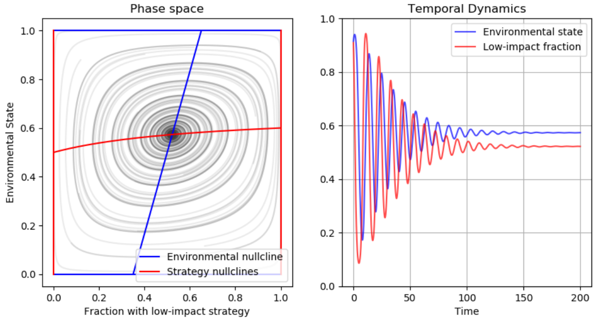

(c) Linear case, fast environmental feedback

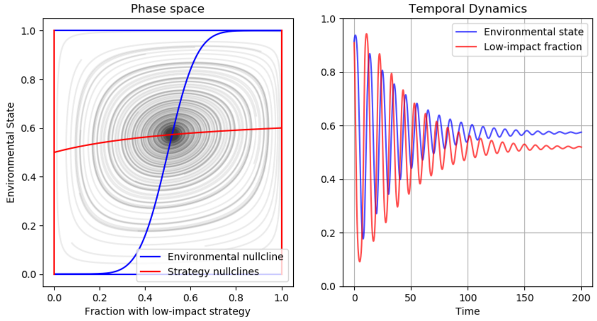

(d) Non-linear case, fast environmental feedback

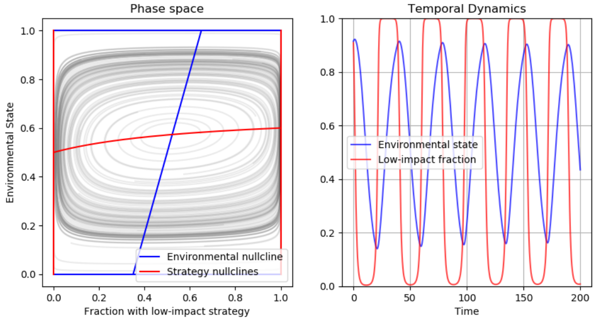

(e) Linear case, slow environmental feedback

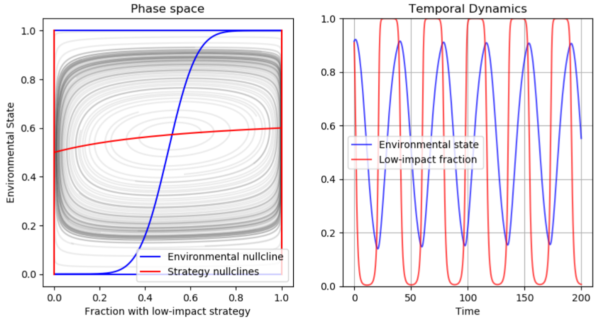

(f) Non-linear case, slow environmental feedback

Supplementary Figure 5: Phase space and temporal dynamics for environmental feedback via environments with tipping points. Panels (a) and (b) show heteroclinic cycles resulting from two models where there is a single environmental tipping point. Panels (c) and (d) show that under a distribution of tipping points, fast environmental feedbacks lead to a stable internal equilibrium for both linear and non-linear cases. Panels (e) and (f) show that for a distribution of tipping points, limit cycles result for slow environmental feedback.

points that define the corners of the state space, where we evaluate the incentives to change strategies, are given by $(x, F) \in \{(0, 1-h/R), (0, 1), (1, 1-h/R), (1, 1)\}$; and the incentives to change strategies are

$$\Delta_L^1 = 1 - c - \xi \tag{154}$$
$$\Delta_H^1 = 1 - c + \xi \tag{155}$$
$$\delta_H^0 = c - 1 + h/R - \xi \tag{156}$$
$$\delta_L^0 = c - 1 + h/R + \xi. \tag{157}$$

Sigdel et al. [5] examine the case where $h/R = 1/2$, $c \in [0, 1]$ and $\xi \in [0, 1]$. When $1/2 < c < 1$ and ξ is small but positive, all the incentive parameters will be positive, with greater incentive to be a follower than a leader of change. Thus we expect this system to fall in the region of possible cycles in Figure 1b. This intuition, gleaned from our simple framework, is confirmed by the existence of cycles in this region in the model of Sigdel et al. [5].

When ξ increases, the incentives to lead (δ_H^0, Δ_L^1) decline, while the incentive to follow (δ_L^0, Δ_H^1) increase. This increases the range of social learning rates, κ, that lead to cyclic dynamics. However, if ξ is sufficiently large then one (and eventually both) of the incentives to lead change (δ_H^0, Δ_L^1) will become negative. This corresponds to a switch from the cycles region of Figure 1b to the single opinion dominance regions of Figure 1c or Figure 1d, and finally, when both incentive to lead become negative, to the bistability region of Figure 1a.

Similar arguments can be made for cases with $h > R$, however, this parameter region falls outside that which we analyzed exhaustively. The relevant corners of the state space shift to $(x, F) \in \{(0, 0), (0, 1), (1, 0), (1, 1)\}$. The incentives to lead and follow change in extreme environments are given by

$$\Delta_L^1 = 1 - c - \xi \tag{158}$$
$$\Delta_H^1 = 1 - c + \xi \tag{159}$$
$$\delta_H^0 = c - \xi \tag{160}$$
$$\delta_L^0 = c + \xi. \tag{161}$$

Although this instance of the model of Sigdel et al. [5] falls outside the cases we studied, the incentive parameters nonetheless paint the same qualitative picture found in Sigdel et al. [5]: the boundaries between the regions where distinct qualitative outcomes occur are defined by the locations where the incentives to lead change (Δ_L^1, δ_H^0) change sign.

Thus the result of Sigdel et al. [5], that strong social norms (large ξ) can eliminate cyclic dynamics, can be understood in terms of how the social norms in their model effect the incentives to lead and to follow change, and how increasing the strength of norms moves the system through the different dynamical regions shown in Figure 1.

Sigdel et al. [6] consider three social-ecological models. The first is closely related to the model discussed above, and it is also a special case of the renewing resource model considered in our analysis. Sigdel et al. [6] also analyzed two other models that are not special cases of the models we have analyzed. Nonetheless, these authors show that there are broad parameter regimes under which these distinct models produce similar patterns. Therefore, the results of our paper may similarly generalize. In particular, they analyze cases where

the environment has Allee effects. This added complexity can create hysteresis, and critical transitions from which recovery is unlikely in the absence of external forcing.

Supplementary References

[1] Joshua S Weitz, Ceyhun Eksin, Keith Paarporn, Sam P Brown, and William C Ratcliff. An oscillating tragedy of the commons in replicator dynamics with game-environment feedback. *Proceedings of the National Academy of Sciences*, 113(47):E7518–E7525, 2016.

[2] David G Rand, Damon Tomlin, Adam Bear, Elliot A Ludvig, and Jonathan D Cohen. Cyclical population dynamics of automatic versus controlled processing: An evolutionary pendulum. *Psychological review*, 124(5):626, 2017.

[3] Alessandro Tavoni, Maja Schlüter, and Simon Levin. The survival of the conformist: social pressure and renewable resource management. *Journal of theoretical biology*, 299: 152–161, 2012.

[4] Andrew R Tilman, James R Watson, and Simon Levin. Maintaining cooperation in social-ecological systems: Effective bottom-up management often requires sub-optimal resource use. *Theoretical Ecology*, 10(2):155–165, 2017.

[5] Ram P Sigdel, Madhur Anand, and Chris T Bauch. Competition between injunctive social norms and conservation priorities gives rise to complex dynamics in a model of forest growth and opinion dynamics. *Journal of theoretical biology*, 432:132–140, 2017.

[6] Ram Sigdel, Madhur Anand, and Chris T. Bauch. Convergence of socio-ecological dynamics in disparate ecological systems under strong coupling to human social systems. *Theoretical Ecology*, 12(3):285–296, Sep 2019. ISSN 1874-1746. doi: 10.1007/s12080-018-0394-z. URL https://doi.org/10.1007/s12080-018-0394-z.
